# Chemical Composition of *Salvia fruticosa* Mill. Essential Oil and Its Protective Effects on Both Photosynthetic Damage and Oxidative Stress in *Conocephalum conicum* L. Induced by Environmental Heavy Metal Concentrations

**DOI:** 10.3390/antiox12111990

**Published:** 2023-11-11

**Authors:** Natale Badalamenti, Giovanna Salbitani, Piergiorgio Cianciullo, Rosanna Bossa, Francesca De Ruberto, Valeria Greco, Adriana Basile, Viviana Maresca, Maurizio Bruno, Simona Carfagna

**Affiliations:** 1 Dipartimento di Scienze e Tecnologie Biologiche, Chimiche e Farmaceutiche (STEBICEF), Università degli Studi di Palermo, Viale delle Scienze Ed. 17, 90128 Palermo, Italy; natale.badalamenti@unipa.it (N.B.); maurizio.bruno@unipa.it (M.B.); 2 NBFC, National Biodiversity Future Center, 90133 Palermo, Italy; 3 Department of Biology, University of Naples Federico II, 80126 Naples, Italy; giovanna.salbitani@unina.it (G.S.); piergiorgio.cianciullo@unina.it (P.C.); rosanna.bossa@unina.it (R.B.); valeriagreco-97@live.it (V.G.); simcarfa@unina.it (S.C.); 4 Department of Clinical Medicine and Surgery, University of Naples Federico II, 80131 Napoli, Italy; francesca.deruberto@unina.it; 5 Centro Interdipartimentale di Ricerca “Riutilizzo Bio-Based Degli Scarti da Matrici Agroalimentari” (RIVIVE), Università degli Studi di Palermo, 90128 Palermo, Italy

**Keywords:** *Salvia fruticosa* Mill., lamiaceae, essential oil, 1,8-cineole, oxidative stress, photosynthetic damage, protection, heavy metals, environmental concentrations

## Abstract

The genus *Salvia* L., belonging to the Lamiaceae family, contains more than 900 species distributed in various parts of the world. It is a genus containing aromatic plants used both in the culinary field and above all in the cosmetic area to produce several perfumes. *Salvia fruticosa* Mill., notoriously known as Greek *Salvia*, is a plant used since ancient times in traditional medicine, but today cultivated and used in various parts of Europe and Africa. Polar and apolar extracts of this plant confirmed the presence of several metabolites such as abietane and labdane diterpenoids, triterpenoids, steroids, and some flavonoids, causing interesting properties such as sedative, carminative, and antiseptic, while its essential oils (EOs) are mainly characterized by compounds such as 1,8-cineole and camphor. The aim of this work concerns the chemical analysis by GC and GC-MS, and the investigation of the biological properties, of the EO of *S. fruticosa* plants collected in eastern Sicily. The gas-chromatographic analysis confirmed the presence of 1,8-cineole (17.38%) and camphor (12.81%), but at the same time, also moderate amounts of *α*-terpineol (6.74%), *β*-myrcene (9.07%), camphene (8.66%), *β*-pinene (6.55%), and *α*-pinene (6.45%). To study the protective effect of EOs from *S. fruticosa* (both the total mixture and the individual compounds) on possible damage induced by heavy metals, an in vitro system was used in which a model organism, the liverwort *Conocephalum conicum*, was subjected to the effect of a mix of heavy metals (HM) prepared using values of concentrations actually measured in one of the most polluted watercourses of the Campania region, the Regi Lagni. Finally, the antioxidant response and the photosynthetic damage were examined. The exogenous application of the EO yields a resumption of the oxidative stress induced by HM, as demonstrated by the reduction in the Reactive Oxygen Species (ROS) content and by the increased activity of antioxidant enzyme catalase (CAT) and glutathione-*S*-transferase (GST). Furthermore, plants treated with HMs and EO showed a higher F_v_/F_m_ (maximal quantum efficiency of PSII in the dark) with respect to HMs-only treated ones. These results clearly indicate the protective capacity of the EO of *S. fruticosa* against oxidative stress, which is achieved at least in part by modulating the redox state through the antioxidant pathway and on photosynthetic damage.

## 1. Introduction

The Lamiaceae family (formerly Labiatae) includes several aromatic plants in all its parts widely cultivated all over the world comprising common herbs such as basil, mint, rosemary, sage, lavender, etc. Since ancient times Lamiaceae have been widely used in the culinary field by peoples like the Romans and the Greeks.

It is a widespread family, comprising more than 220 genera and 4000 species of annuals and perennial plants. A huge number of papers have been published on their chemistry concerning both the essential oils (EOs) (mainly monoterpene and sesquiterpene compounds), and the non-volatile constituents such as diterpenes (labdane, abietane, and clerodane diterpenes), triterpenes, and phenolics as well as on their ethno-pharmaceutical and biological properties [1,2]. 

*Salvia* L., the largest genus of this family, includes more than 900 species and is divided into five subgenera (*Sclarea*, *Audibertia*, *Jungia*, *Leonia,* and *Salvia*). Many species of this genus are used, due to their colored flowers, typically pink to red or purple to blue, as ornamental plants, whereas other ones have economic importance since they are utilized as flavoring agents in perfumery and cosmetics. The name “Salvia” (sauge in French and sawge in old English) derives from the Latin word “salvare” meaning “to heal or to be safe”, due to the folkloric belief of its “magical” therapeutic properties and its diffusion in popular medicine because of diverse biological activities, including antibacterial, spasmolytic, hemostatic, and many others [3].

The genus *Salvia* has a sub-cosmopolitan distribution, widely present in many regions of the world including the warmer and temperate zones of the world such as the Mediterranean, Central Asia, Pacific Islands, tropical Africa, and America, with the largest number of species (about 300) occurring in Mexico [4,5]. 

*Salvia fruticosa* Mill. (syn. *S. triloba* L., *S. libanotica* Boiss. and Gaill., *S. lobryana* Azn., *S. cypria* Unger and Kotschy, etc.), commonly known as Greek sage, is a perennial shrub native to Eastern Mediterranean including Southern Italy, Sicily, southern parts of the Balkan Peninsula to West Syria, Cyprus, and Libya [6]. It grows on dry rocky limestone soils or the edges of pine forests, riverbeds, and roadsides, at altitudes from 100 to 800 m a.s.l and it is common and abundant in plant communities of garigue in the eastern Mediterranean region, associated with *Sarcopoterium spinosum*, *Micromeria nervosa*, *Cistus creticus*, *Cistus salvifolius*. Due to the value of its EOs, it has been introduced in the Western Mediterranean region in Algeria, Morocco, Malta, Spain, and Portugal [7]. 

The use of *S. fruticosa* probably dates to 1400 B.C. as shown in the “blue bird fresco” in the House of Frescoes, Knossos, and its peculiar shape with opposed trilobate leaves was also represented on Iberic pottery, under Greek influence (400 B.C.). It has been used in folk medicine since ancient times [7].

The infusion of its leaves is extremely popular among the Palestinians of Israel for relieving headaches and in the treatment of rheumatism, heart disorders, stomach, abdominal, and ulcer pains, or for indigestion [8,9]. For similar purposes, to treat rheumatic pains, the infusion of leaves and tender shoots is used in North Africa [10]. In the markets of Jordan, it is also sold as a sedative, carminative, and stomachic, as an antiseptic and vulnerary [11]. In Turkey, this species is used for poor appetite stomachache, kidney, and gallbladder stones, and sands [12]. In Antalya, Turkey [12], North Africa [10], and among the Arabs of Israel [8,9] the infusion of leaves and young shoots of *S. fruticosa* is employed for colds, coughs, and influenza. Its use as a vulnerary and cicatrizing has been reported in Murcia and Israel, and it was cited in the ancient literature as hemostatic, cicatrizing, antiseptic, a remedy for sore throat, and anti-pruriginous effects [7]. The dried leaves are utilized to make a tea “faskomelo” that is sold in cafes in Greece and Turkey [13]. The fresh leaves are also infused with sugar or honey [13] and are reputed as medicinal in Greece. Several similar uses of this plant have been reported for Spain and Portugal [7]. 

Most of the health effects of *S. fruticosa* are attributable to the presence of bioactive substances that are metabolized by the body with the aid of extracts and infusions [14]. Usually, this aspect is attributable to the presence of active principles of an antioxidant nature such as polyphenols that influence the antioxidant state or modulate critical enzymes [15]. Due to all these beneficial properties, *S. fruticosa* is accepted as a medicinal *Salvia* species by the European Pharmacopeia and British Pharmacopeia [16]. 

From the aerial parts of *S. fruticosa,* several non-volatile metabolites have been isolated, including abietane diterpenoids, labdane diterpenoids, triterpenoids, steroids, and several flavonoids [17,18,19,20]. 

Many papers have been published on the EOs of different accessions of *S. fruticosa* (Table 1), but no one concerns the Sicilian population. Consequently, as a continuation of our research on plants of the Mediterranean area [21,22,23,24] and on the biological properties of EOs [23,25,26], the present paper reported on the EO composition of the aerial parts of *S. fruticosa,* collected in Sicily, as well the biological properties of the EO obtained from the full-flowering aerial parts. 

The ability of EOs to exert a protective effect against damage from heavy metals in plant organisms is a topic of recent research and is still little studied. It has recently been demonstrated that the EO of *Thymus leucotrichus* can reduce Cd toxicity in the aquatic moss *Leptodictyum riparium* [24]. In particular, was demonstrated that the exogenous application of the EO yields a resumption of growth rate and a reduction in the number of dead cells; it also reduces the oxidative stress induced by Cd, as demonstrated by the reduction in the Reactive Oxygen Species (ROS) content (with a decrease of 1.52% and 5%) and by the increased activity of antioxidant enzymes such as superoxide dismutase (SOD) (with an increase of 1.44% and 2.29%), catalase (CAT) (1.46% and 2.91%), and glutathione-*S*-transferase GST (1.57% and 1.90%). Furthermore, the application of the EO yields a reduction in DNA damage. 

At the moment, however, there is no work in which the protective effect of EOs is studied with respect to damage caused by concentrations actually measured in a polluted environment. For this reason, it was decided to study the protective effect of the EO extracted from *S. fruticosa*, on another model organism, however, belonging to the Bryophyte group, the liverwort *Conocephalum conicum*, using a mix of heavy metals at the concentrations measured in two sites with high anthropic impact, chosen along the course of the Regi Lagni. 

The Regi Lagni consists of a network of straight channels that collect meteoric, spring and, also, waste waters, carry them from the plain north of Naples to the Tyrrhenian Sea, covering a length of about 56 km [71]. The Regi Lagni basin has been declared a National Concern Site (NCS) by the Italian Government because of its huge contamination potential being in a completely careless condition and affected by severe contamination caused by heavy urbanization and industrialization (mainly chemical industry) as well as intensive agriculture and buffalo farms [71,72,73].

The model organism chosen is a bryophyte, as, unlike other terrestrial plants, it absorbs water, mineral salts, and all that is present in the environment, through the entire surface of the body, and is, therefore, entirely and inexorably subject to all its cells, to the positive or negative effects of all that is present in the solutions with which it comes into contact.

Furthermore, *C. conicum* is a plant often used in studies on the damage and metabolic responses induced by heavy metals because is a cosmopolitan species able to respond to local environmental pollution by changing its biological features. 

The aim of this work is to study the protective effect of EO from *S. fruticosa* on possible damage induced by heavy metals. In vitro, the liverwort *C. conicum*, was subjected to the effect of a mix of heavy metals prepared using values of concentrations measured in two sites chosen along the Regi Lagni channels. To assess the protective effects, the photosynthetic damage and the antioxidant response were examined, with or without the exogenous application of the total mixture and the individual compounds. In particular, as for oxidative stress protection, ROS content and activity of antioxidant CAT and glutathione-*S*-transferase (GST) were measured. Regarding the protective capacity of the EO of *S. fruticosa* on photosynthetic damage, F_v_/F_m_ (maximal quantum efficiency of PSII in the dark) with respect to HMs only treated ones, was considered.

## 2. Materials and Methods

### 2.1. Essential Oil Extraction

The flower in aerial parts of *S. fruticosa* was collected near Noto Antica, Syracuse, (Sicily, Italy) (36°57′27.37″ N; 15°02′18.76″ E) at 378 m a.s.l., on 12 June 2022, and a voucher specimen has been deposited in the STEBICEF Department, University of Palermo, Italy (PAL1135510). The fresh aerial parts (160 g) of *S. fruticosa* were subjected to hydrodistillation for 3 h using Clevenger’s apparatus [74]. The EO, yielding 1.2% (*w/w*), was dried with anhydrous sodium sulfate, filtered, and stored in the freezer at −20 °C, until the time of analysis.

### 2.2. Gas Chromatography–Mass Spectrometry (GC–MS) Analysis of Essential Oil 

Analyses of EOs were performed according to the procedure reported by Rigano et al. [75]. Analysis of EO was performed by a Shimadzu QP 2010 plus equipped with an AOC-20i autoinjector and an apolar capillary column, DB-5 MS, 30 m × 0.25 mm i.d., film thickness 0.25 μm and a data processor (GCsolution software v. 2.53, Shimadzu, Kyoto, Japan). The other column used was the polar Supelcowax 10 (Merck KGaA, Darmstadt, Germany) which had the same with the same length and thickness as the previous one listed here. The oven program was as follows: initial temperature 40 °C for 5 min, from 40 °C to 260 °C at a rate of 2 °C/min, then isothermal for 20 min. Helium was used as carrier gas (1 mL min^−1^). The injector and detector temperatures were set at 250 °C and 290 °C, respectively. The 1 μL of EO solution (3% EO/hexane *v/v*) was injected with split mode 1:10. The percentages are calculated by individually integrating the peak areas in the chromatogram. The analyses were performed in triplicate and the results are expressed as the average of three measurements ± standard deviation. Linear retention indexes (LRIs) were determined by using retention times of *n*-alkanes (C_8_-C_40_) and the peaks were identified by comparison with mass spectra with WILEY275, NIST 17, ADAMS, and FFNSC2 libraries.

### 2.3. Conocephalum Conicum Material 

Samples of *C. conicum* L. Dum were collected in March 2020 from upstream of the Regi Lagni, identified by Prof. Adriana Basile and a sample was deposited in the herbarium of the Botanical Garden of the University Federico II Napoli (NAP 986-216). These samples were used for in vitro experiments.

### 2.4. In Vitro Growth 

The samples of *C. conicum* were placed in Petri dishes (diameter 10 cm) after a careful but delicate removal of the layer of soil adhering to the lower surface held by the rhizoids, with a small brush, so that Mohr’s solution, pH 7,5 [76], wetted only the lower portion of the thallus. It was important not to “submerge” the samples to ensure that the plant was able to carry out gas exchange through the pores correctly.

The soluble salts CdCl_2_, CuSO_4_, Pb(CH_3_COO)_2_, and ZnCl_2_ were added to Mohr’s medium. In the control Mohr’s mediums Cl^−^ and SO_4_ anions were added instead as K salts (KCl, K_2_SO_4_) to maintain the same concentrations as the exposure solutions. The heavy metal concentrations used are those measured at the field sites, indicated below as C1 upstream site (40°49′56.269″ N, 14 °35′27.103″ E), C2 downstream site (40°44′48.812″ N, 14°31′37.653″ E). The concentrations used are those reported in the study by Maresca et al., 2018 [77] and are shown in Table 2. The cultures were maintained for 7 days in a climatic room and the environmental parameters were set according to the environmental conditions registered in the field. In particular, the air temperature was maintained at 20.0 ± 1.5 °C, and 13.0 ± 0.7 °C, mean ± SD, during day and night, respectively; relative humidity was 70 ± 4% mean ± SD, 16 h light (Photosynthetic Active Radiation 400 μmolm^−2^ s^−1^)/8 h dark photoperiod. These environmental parameters were chosen according to the period of the year in which the collection took place so as not to subject the samples to further stress. 

### 2.5. Treatment with the Total Extract of EO and the Individual Compounds

To test the effects of EO of *S. fruticosa*, the *C. conicum* samples were treated both with the total EO and with the single molecules present in higher percentages, namely 1,8-cineole, camphor, and *β*-myrcene.

Samples were treated with both total EO and single compounds at concentrations of 0.16% and 0.25% (*v*/*v*) as a spray for 7 days. The EO and individual compounds were dissolved in 5% dimethyl sulfoxide (DMSO) followed by dilution with water containing the surfactant Tween 20 (0.1%, *v*/*v*). Each sample was sprayed simultaneously every day for 7 days. Treatments and sample names are shown in Table 3.

### 2.6. Detection of ROS and Antioxidant Enzymes’ Activity

For the quantitative measurement of ROS production, 2′,7′-dichlorofluorescine diacetate (DCFH-DA) was used following the protocol reported in [77] Maresca et al., (2018). The amount of ROS was monitored by fluorescence (excitation wavelength of 350 nm and emission wavelength of 600 nm) using a multiplate reader (Synergy H4, Agilent Technologies, Inc., Santa Clara, California, USA).

CAT activity (Units (CAT) mg proteins^−1^) was kinetically measured (for 1 min at 25 °C, 15 s each read) as a decrease in the absorbance of H_2_O_2_ at 240 nm in 2 mL quartz cuvettes with a spectrophotometer UV-Vis (Cary 300, Agilent Technologies, Inc.) using a commercial kit (Sigma–Aldrich Co., St Louis, MO, USA). and The drop in absorbance at 240 nm is linear with the consumption of H_2_O_2_ and was used to quantify the umol H_2_O_2_ consumed (ε = 0.0436 mM^−1^, path length = 1 cm). By definition one unit of catalase is defined as the unit able to decompose 1.0 µmole of H_2_O_2_ per minute at pH 7.0 at 25 °C, and the CAT units in the samples were calculated accordingly. Glutathione *S*-transferase (GST, EC 2.5.1.18) activity was measured using a commercial kit (CS0410, Sigma). The reactions were monitored for 6 min at 25 °C using a multiplate reader (Synergy H4, Agilent Technologies, Inc.). The increase in absorbance at 340 nm that indicates the conjugation of reduced glutathione with the 1-chloro-2,4-dinitrobenzene (CDNB) was recorded and the umol of CDNB-GSH conjugates was quantified according to their molar extinction coefficient (ε = 5.3 mM^−1^, path length = 0.552 cm).

The quantification of total soluble proteins was carried out with Bradford assay (Bio-rad Laboratories, Inc., Hercules, California, U.S.A.) using bovine seroalbumin to calibrate the standard curve. Each assay was run in triplicate for each sample (N = 3)

### 2.7. Measurements of Chlorophyll Fluorescence

To define the photosynthetic capacity in the control and treated plants, samples were analyzed with a Maxi Imaging-PAM M-Series Chlorophyll Fluorometer (Heinz Walz GmbH, Effeltrich, Germany). Plants were acclimated in the dark for 30 min before analysis. After dark adaptation, the maximal quantum efficiency of PSII in the dark (F_v_/F_m_, where F_v_ is the variable and F_m_ is the maximal fluorescence in dark-adapted organisms) was measured. Regarding F_v_/F_m_, samples were illuminated with a saturating pulse, as reported in [78], and values derived from the formula F_v_/F_m_ = (F_m_ − F_0_)/F_m_.

## 3. Results and Discussion

### 3.1. Chemical Profiling of Salvia Fruticosa EO

Hydro-distillation of the aerial parts of *S. fruticosa,* in full flowering period, gave an intense-yellow EO with a yield of 1.2% (*w*/*w*). Overall, forty-one compounds were found, representing 93.27% of the total composition. In Table 4, according to their linear retention indices on a DB-5 MS column, the components are listed and classified based on their chemical structures into eight different classes, the principal ones being monoterpene hydrocarbons (31.60%), and oxygenated sesquiterpenes (49.11%).

This EO was quite rich in 1,8-cineole (eucalyptol) (17.56%), and camphor (13.63%), both belonging to the oxygenated sesquiterpenes class. In the same group, it is worthy of mention the occurrence of a good quantity of *α*-terpineol (6.56%). Monoterpene hydrocarbons were characterized by the presence, in similar amounts, of four metabolites: *β*-myrcene (9.13%), camphene (8.69%), *β*-pinene (6.70%), and *α*-pinene (6.51%). Oxygenated sesquiterpenes (5.58%) were mainly represented by globulol (4.07%), whereas manool (3.01%) was the principal constituent of the oxygenated diterpenes (3.18%). These percentages are calculated assuming that the total of the compounds found is 100%.

The aspect that emerged from the GC-MS analysis of the EO collected in Sicily is the total similarity with the other EOs isolated in different parts (Greece, Israel, Lebanon, and Turkey) of the world from both cultivated and wild species. Compounds 1,8 cineole, camphor, *α*-pinene, and *β*-pinene are compounds present in almost all the EOs scientifically analyzed and published in the literature. Diversities emerged for compounds such as *α*- and *β*-thujone, ketone monoterpenes, sometimes present in high and modest quantities (samples of Albania, Brazil, Greece, and Turkey) and often absent or in minimal concentrations in various specimens (Cyprus, Italy, and Jordan).

### 3.2. ROS Quantificaztion and Antioxidant Enzymes

**Figure 1:**
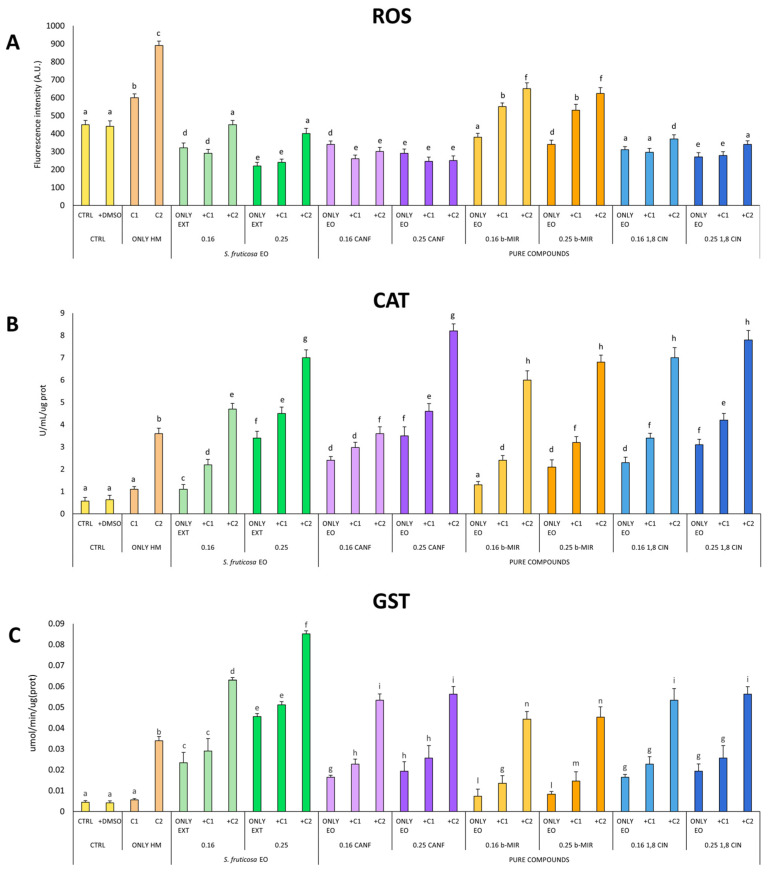
ROS production (**A**), CAT, U/mL/mg of protein (**B**) and GST, umol/min/ug(prot) (**C**) in *C. conicum* samples. Bar marked with different letters are statistically different for ANOVA Tukey’s Post-hoc test (*p* < 0.05).

The production of ROS and the activation of catalase and glutathione-S-transferase were measured in the C. conicum gametophytes. Control and control-DMSO samples show the basal ROS signal (fluorescence intensity A.U.) produced by the basal cellular metabolism in the liverwort and other plants and participate in fundamental biological processes such as cell signaling, development, environmental stimuli, metabolism, etc. [79] (Figure 1A). Yet in C1 exposed gametophytes an increase in ROS was observed, with a clear and wider increase in C2 exposed samples. Alongside ROS increases, the enhancement of CAT and GST activities was observed. CAT and GST both participate in the enzymatic antioxidant defenses to balance the outburst of ROS in stress conditions e.g., during heavy metal exposure [80,81].

The application of the Salvia fruticosa total EO lowered the basal ROS as shown in CTRL-TE16 and CTRL-TE25 (Figure 1A) and enhanced both CAT and GST activities (Figure 1B,C) in a dose-dependent manner. The treatment of the C1 and C2 exposed gametophytes with EO (C1-C2TE16 and C1-C2TE25) significantly decreased ROS production compared to untreated samples (C1 and C2; Figure 1A).

The most abundant compounds of EO (camphor, β-myrcene, and 1,8 cineole) were tested separately to dissect their contribution to prevent ROS outbursts and enhance the enzymatic antioxidant response. As shown in Figure 1B,C camphor and 1,8 cineole induced CAT and GST activities to a greater extent with respect to β-myrcene thus aiding in lowering ROS production (Figure 1A). However, also the application of β-myrcene had the effect of augmenting CAT and GST activities and lower ROS outburnst, by inducing CAT and GST (CTRL-MYR16 and 25; Figure 1B,C).

### 3.3. Measurements of Chlorophyll Fluorescence

**Figure 2:**
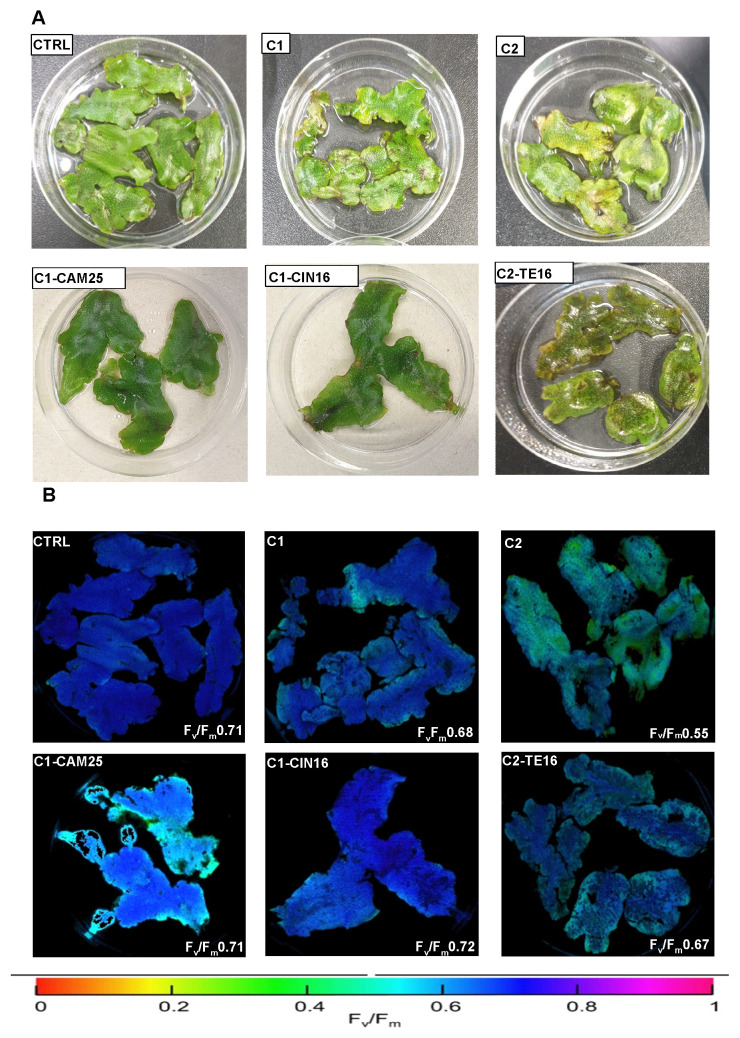
(**A**): Pictures of control and some of more significant samples of *C. conicum*, after 7 days of treatment. (**B**): Maximal quantum efficiency of photosystem II (F_v_/F_m_) after 7 days of treatment, obtained by Imaging-PAM. F_v_/F_m_ values of treated samples (C1, C2, C1-CAM25, C1-CIN16, C2-TE16) and not (CTRL) were showed bottom right of the panels. The false-color scale indicates the F_v_/F_m_ values and range from black (0.0) to purple (1.0) is shown.

Samples of *C. conicum* were exposed to HM solutions (C1 and C2) in the presence or absence of different concentrations of EO (0.16% and 0.25%). Figure 2 shows a representative result of EO treatment imposed on HM-stressed plants. The complete EO application resulted in a significative F_v_/F_m_ improvement on C1-CAM25 and C1-CIN16 with respect to only C1 HM treated. Indeed, in C2 the exposure to HM reduced the F_v_/F_m_ by 25%, while the application of EO on samples C2-TE16 improved the chlorophyll fluorescence that maintaining values close to those of the CTRL. The results obtained by Imaging-PAM demonstrated a positive protection effect of EO on photosynthetic efficiency of photosystems II. In addition, the images captured by Imaging-PAM (Figure 2B) show the presence of not homogeneous damage occurring especially on leaves margins. This result depends on the natural curled shape of leaves that prevents the homogenous touch with the media.

## 4. Conclusions

In conclusion, the analytical chemical analysis by GC-MS of the EO of spontaneous Sicilian plants of *S. fruticosa* revealed the presence of bioactive compounds such as eucalyptol (17.38%), and camphor (12.81%), oxygenated sesquiterpenes class, and at the same time moderate amounts of monoterpenes such as *α*-terpineol (6.74%), *β*-myrcene (9.07%), camphene (8.66%), *β*-pinene (6.55%), and *α*-pinene (6.45%). The treatment of *C.conicum* with some EO relieves or avoids the photosystem damage due to HM exposure, allowing plants to maintain a good photosynthetic performance. This work shows preliminary data about a possible application of EO to enhance the efficiency of plants in phytoremediation processes.

## Figures and Tables

**Table 1:** Main constituents (>3%) of the essential oils (EOs) of *Salvia fruticosa*, obtained by hydro-distillation, reported in literature.

Origin	P.p.	Compounds (%)	Ref.
Albania	l.	1,8-cineole (38.9), camphor (8.4), *α*-pinene (5.9), *α*-thujone (5.4), *α*-terpineol (4.9), *β*-pinene (4.4), borneol (3.8), camphene (3.8), (*E*)-*β*-caryophyllene (3.5), *β*-thujone (3.4), myrcene (3.2)	[26]
Albania, 7 localities	l.	1,8-cineole (51.2–17.2), camphor (18.6–2.9), (*E*)-*β*-caryophyllene (16.0–0.7), *β*-thujone (10.4–1.1), *α*-pinene (6.0–1.7), camphene (6.0–0.5), *α*-terpineol (5.7–0), globulol (5.0–0), *β*-pinene (4.5–2.2), manool (4.5–0.8), *α*-terpenyl acetate (4.2–0), *α*-thujone (4.1–0.9)	[27]
Albania, Vlora	a.p.	1,8-cineole (37.5–30.1), camphor (21.5–13.9), camphene (9.0–6.4), (*E*)-*β*-caryophyllene (8.1–5.3), *α*-pinene (7.1–6.6), *β*-pinene (5.6–4.5), myrcene (5.5–4.0)	[28]
Brasil	a.p.	*α*-thujone (20.1), 1,8-cineole (15.7), camphor (12.6), (*E*)-*β*-caryophyllene (11.8), *α*-humulene (7.5), viridiflorol (6.3), *β*-thujone (4.8), *β*-pinene (3.9)	[29]
Cyprus, Troodos	l.	camphor (49.3–49.2), 1,8-cineole (21.5–17.6), (*E*)-*β*-caryophyllene (11.9–6.6), camphene (5.0–0), borneol (4.6–1.7), limonene (3.6–0)	[30]
Cyprus, 6 localities	l.	1,8-cineole (67.5–19.3), camphor (44.5–5.7), camphene (7.3–1.4), *β*-pinene (6.9–2.2), limonene (5.3–1.1), *α*-pinene (4.3–3.3)	[31]
Cyprus, 4 localities	f.	1,8-cineole (52.0–14.3), camphor (41.8–6.3), *β*-pinene (13.9–3.0), (*E*)-*β*-caryophyllene (8.7–4.4), camphene (6.5–2.1), *α*-pinene (5.7–1.8), borneol (5.2–3.5), limonene (3.1–1.6)	[32]
Cyprus, 5 localities	s.	1,8-cineole (54.7–4.0), camphor (44.2–7.6), (*E*)-*β*-caryophyllene (23.0–3.4), caryophyllene oxide (12.2–1.9), *β*-pinene (9.7–0), borneol (7.6–1.5), camphene (6.5–0), *α*-pinene (4.2–0)	[31]
Greece, 3 localities	l.	1,8-cineole (58.3–23.7), globulol (9.9–0), *β*-thujone (9.8–2.6), *α*-terpineol (6.4–3.2), manool (6.4–0), *β*-pinene (6.1–0.8), *α*-terpenyl acetate (5.2–0), *α*-pinene (4.2–0.4), (*E*)-*β*-caryophyllene (3.8–0.8), *α*-thujone (3.5–1.2),	[27]
Greece, 15 localities	l.	1,8-cineole (54.4–16.9), (*E*)-*β*-caryophyllene (15.6–0), camphor (15.4–0.6), *α*-thujone (14.5–0), *β*-thujone (9.0–0.6), *β*-pinene (9.0–0), viridiflorol (8.4–0), borneol (8.0–0), *α*-pinene (7.4–1.5), camphene (7.0–0), bornyl acetate (6.8–0), *α*-terpineol (6.7–0), myrcene (5.2–1.6)	[32]
Greece, 8 localities	l.	1,8-cineole (66.2–38.8), camphor (23.8–1,7), thujone (12.1–1.4), *β*-pinene (10.7–2.9), camphene (7.4–0.4), (*E*)-*β*-caryophyllene (7.3–1.2), *α*-pinene (6.7–3.7), myrcene (6.7–0)	[33]
Greece, Ikaria	a.p.	camphor (23.1), *α*-pinene (12.7), borneol (12.6), camphene (9.0), 1,8-cineole (6.9), *β*-pinene (5.8), (*E*)-*β*-caryophyllene (5.3), *α*-terpineol (4.6), caryophyllene oxide (3.8)	[34]
Greece, Kalymnos	a.p.	1,8-cineole (31.4), camphor (22.6), *α*-pinene (8.7), camphene (8.5), *α*-thujone (7.5), *β*-pinene (4.5), *β*-thujone (4.1)	[34]
Greece, Krete	a.p.	1,8-cineole (41.4), camphor (12.1), *β*-thujone (10.3), *β*-pinene (6.4), *α*-pinene (5.4), *α*-terpineol (5.0), camphene (3.1)	[35]
Greece, Krete	a.p.	1,8-cineole (64.2–22.7), camphor (30.3–0.8), *β*-thujone (25.6–0.9), *α*-thujone (19.2–1.0), camphene (9.9–0.2), *β*-pinene (9.4–3.5), *α*-terpineol (7.5–1.2), (*E*)-*β*-caryophyllene (6.9–0.2), myrcene (5.3–1.6), *α*-pinene (5.2–1.8),	[36]
Greece, Krete	l.	1,8-cineole (51.0–35.6), camphor (11.6–3.7), *β*-thujone (11.5–1.9), *β*-pinene (7.0–5.0), *α*-thujone (5.6–2.9), (*E*)-*β*-caryophyllene (4.7–1.3), *α*-pinene (4.5–3.6), camphene (3.2–0.7)	[37]
Greece, Krete, clt NO_3_-N, 100 mg/L	l.	1,8-cineole (37.5–26.8), viridiflorol (15.7–7.2), (*E*)-*β*-caryophyllene (13.0–0.2), 13-*epi*-manool (11.4–4.6), myrcene (7.0–3.7), *α*-humulene (6.0–4.8), *α*-pinene (5.1–3.7), *β*-pinene (4.6–4.5), *α*-aromadendrene (4.5–3.1), *α*-terpineol (3.6–2.5)	[38]
Greece, Krete, clt NO_3_-N, 150 mg/L	l.	1,8-cineole (28.6–22.5), 13-*epi*-manool (13.1–12.9), (*E*)-*β*-caryophyllene (12.2), viridiflorol (10.9–10.7), *α*-humulene (4.9–4.7), *α*-pinene (4.1–2.6), *β*-pinene (4.0–3.7), *α*-terpineol (3.8–3.0), myrcene (3.7–2.6), *α*-aromadendrene (3.6–3.0)	[38]
Greece, Krete, clt NO_3_-N, 200 mg/L	l.	viridiflorol (37.9–23.3), 13-*epi*-manool (25.7–14.3), (*E*)-*β*-caryophyllene (11.5–9.9), *α*-humulene (10.2–8.6), *α*-terpineol (6.6–6.2), caryophyllene oxide (3.9–3.3)	[38]
Greece, Krete, clt	l.	1,8-cineole (59.3–48.1), *β*-pinene (11.9–10.3), *α*-pinene (10.0–9.3), myrcene (7.8–3.7), camphor (5.9–1.3), *β*-thujone (4.5–0.5)	[39]
Greece, Krete, clt	l.	1,8-cineole (62.9–28.2), *α*-thujone (34.1–2.0), camphor (10.3–0.4), *β*-pinene (8.8–0.9), *β*-thujone (8.6–0.9), (*E*)-*β*-caryophyllene (5.6–1.4), myrcene (5.2–1.1), caryophyllene oxide (5.2–0.2), *α*-pinene (3.9–0.2)	[40]
Greece, Peloponnese	l.	1,8-cineole (46.6–27.8), camphor (15.6–6.2), (*E*)-*β*-caryophyllene (9.7–4.0), camphene (7.4–2.5), *α*-pinene (7.1–4.1), *β*-pinene (5.4–3.3), myrcene (5.4–3.1), *α*-terpineol (4.0–2.0), *β*-thujone (3.0–0.6)	[41]
Greece, clt	a.p.	1,8-cineole (55.7–44.7), camphor (14.9–1.3), *β*-pinene (14.1–5.8), (*E*)-*β*-caryophyllene (7.2–1.4), camphene (5.9–0.5), *α*-pinene (5.9–3.5), myrcene (5.6–2.7), *α*-terpineol (5.2–2.1),	[42]
Greece, clt	a.p.	camphor (18.6), 1,8-cineole (16.6), camphene (7.0), (*E*)-*β*-caryophyllene (5.4), *β*-pinene (5.3), *α*-pinene (5.2), bornyl acetate (4.4), *α*-terpineol (3.9), *α*-thujone (3.8), *β*-thujone (4.1), limonene (3.1)	[43]
Greece, Mt. Ochi, Eubea	a.p.	1,8-cineole (56.3), *β*-pinene (7.8), (*E*)-*β*-caryophyllene (7.0), *α*-terpineol (5.6), *β*-thujone (4.1), *α*-pinene (4.0), myrcene (3.0)	[43]
Greece, Sithonia	l.	1,8-cineole (43.1), camphor (18.3), *β*-pinene (8.2), *α*-pinene (6.8), sabinene (4.8), myrcene (3.2),	[44]
Greece, Zakynthos	a.p.	1,8-cineole (58.9–46.0), viridiflorol (7.0–2.1), camphor (5.8–07), (*E*)-*β*-caryophyllene (5.1–1.0), *β*-pinene (5.0–2.0), myrcene (4.6–3.2), *α*-terpineol (4.3–2.8), *α*-pinene (4.0–3.2), *α*-thujone (3.1–1.1)	[45]
Egypt, 3 localities	a.p.	camphor (23.7–5.1), 1,8-cineole (45.7–31.9), (*E*)-*β*-caryophyllene (11.5–0.9), *β*-pinene (9.9–6.7), camphene (8.7–1.9), *α*-pinene (5.7–2.9), myrcene (4.0–1.6)	[46]
Hungary, clt	a.p.	camphor (26.0), *α*-thujone (21.4), 1,8-cineole (16.9), viridiflorol (5.6), myrcene (4.3)	[47]
Israel, clt	a.p.	1,8-cineole (26.4), camphor (18.9), camphene (9.5), *α*-thujone (9.1), (*E*)-*β*-caryophyllene (5.0), *α*-humulene (3.9), *β*-pinene (4.7), *α*-pinene (4.4)	[48]
Israel, clt	l.	1,8-cineole (44.0), *α*-pinene (18.6), (*E*)-*β*-caryophyllene (11.3), *β*-pinene (5.0), camphor (3.3)	[49]
Israel, clt	s.	*α*-pinene (37.3), 1,8-cineole (31.5), (*E*)-*β*-caryophyllene (7.6), *β*-pinene (7.0), camphor (6.8)	[49]
Israel, clt	f.	*α*-pinene (31.5), 1,8-cineole (30.8), (*E*)-*β*-caryophyllene (10.4), *β*-pinene (6.6), camphor (5.6), *α*-terpinil acetate (3.4), camphene (3.1)	[49]
Italy, Salento, clt	a.p.	1,8-cineole (27.6), (*E*)-*β*-caryophyllene (18.3), limonene (8.8), humulene (7.6), myrcene (5.0), *α*-pinene (3.7), *γ*-gurjunene (3.7)	[50]
Jordan, Amman	l.	1,8-cineole (45.2), camphor (11.5), *β*-pinene (9.0), *γ*-terpineol (4.4), *α*-pinene (3.3)	[51]
Lebanon, Ebrine	a.p.	1,8-cineole (33.5), *β*-pinene (9.8), *α*-pinene (8.0), (*E*)-*β*-caryophyllene (7.6), *α*-thujone (7.1), *α*-terpineol (6.4), camphor (5.6), *α*-terpinyl acetate (3.7), myrcene (3.5)	[52]
Lebanon	a.p.	1,8-cineole (57.3), (*E*)-*β*-caryophyllene (8.3), camphor (4.8), *α*-terpineol (4.2)	[53]
Lebanon	a.p.	1,8-cineole (21.5), *β*-pinene (10.1), *α*-terpineol (9.2), (*E*)-*β*-caryophyllene (7.3), camphor (6.3), camphene (5.0), *γ*-gurjunene (4.4)	[54]
Lebanon, Nahr Ibrahim	a.p.	1,8-cineole (48.7), (*E*)-*β*-caryophyllene (30.8), aromadendrene (3.3), *β*-pinene (3.2)	[55]
Lybia, Biadda	a.p.	1,8-cineole (49.3), camphor (7.5), *β*-pinene (7.4), myrcene (7.4), *α*-pinene (5.1), (*E*)-*β*-caryophyllene (4.1), *α*-terpineol (3.2)	[56]
Turkey, cultivated	a.p.	1,8-cineole (45.0), camphor (7.0), (*E*)-*β*-caryophyllene (5.7), *β*-pinene (5.3), *β*-thujone (5.1), *α*-pinene (5.0), camphene (3.0)	[57]
Turkey, ÇakIroluk	a.p.	1,8-cineole (11.6), camphor (10.4), *α*-thujone (10.4), *β*-gurjunene (8.2), *α*-humulene (7.5), *β*-thujone (4.8), *β*-pinene (3.9)	[58]
Turkey, Iskilip, Çorum	a.p.	1,8-cineole (40.0), camphor (11.3), *α*-pinene (7.3), myrcene (4.5), camphene (3.9)	[59]
Turkey, Izmir, cultivated	a.p.	1,8-cineole (57.2), *β*-pinene (8.2), myrcene (5.7), (*E*)-*β*-caryophyllene (4.8), *α*-pinene (3.4), camphor (3.1), *β*-thujone (3.1)	[60]
Turkey, Konya market	l.	1,8-cineole (51.2), *α*-thujone (5.8), *α*-pinene (4.4), *β*-pinene (3.1)	[61]
Turkey, Kalkan	l.	1,8-cineole (456 mg/mL), thymol (39 mg/mL), camphor (36 mg/mL), *α*-pinene (27 mg/mL), *β*-pinene (20 mg/mL)	[61]
Turkey, Konya, clt	a.p.	1,8-cineole (36.2), camphor (19.1), thujone (7.8), *β*-pinene (6.4), *α*-pinene (5.3), (*E*)-*β*-caryophyllene (4.8), *α*-terpineol (3.9)	[62]
Turkey, Marmara	a.p.	1,8-cineole, (52.8), camphor (5.8), *α*-pinene (5.8), *β*-pinene (4.5), myrcene (3.8), camphene (3.1)	[63]
Turkey, Mersin	a.p.	*α*-pinene (31.0), isoborneol (27.2), borneol (7.6), 1,8-cineole, (6.9), camphene (6.1), *β*-pinene (3.9)	[64]
Turkey, Muğla	a.p.	1,8-cineole (58.9), *α*-pinene (5.6), *β*-pinene (5.2), myrcene (5.2), camphor (4.5), (*E*)-*β*-caryophyllene (4.2), *α*-terpineol (3.0)	[65]
Turkey, Muğla	a.p.	1,8-cineole (55.5), camphor (8.4), (*E*)-*β*-caryophyllene (5.2), borneol (4.6), *β*-pinene (4.3), *α*-pinene (3.2), myrcene (3.1)	[66]
Turkey, Muğla	a.p.	1,8-cineole (40.1), camphor (26.8), borneol (8.9), camphene (5.3), *α*-pinene (3.6)	[67]
Turkey, West Mediteraean	a.p.	1,8-cineole (49.5), camphor (13.3), *β*-pinene (7.2), *α*-pinene (5.8), camphene (5.0), *β*-thujone (3.6)	[68]
Turkey, 3 localities	l.	1,8-cineole (47.1–27.2), camphor (19.8–9.3), camphene (10.7–3.8), *α*-pinene (7.1–5.7), *β*-pinene (5.8–5.7), borneol (4.4–1.5), *α*-thujone (3.4–1.9), (*E*)-*β*-caryophyllene (3.1–1,5)	[69]
Turkey, commercial	l.	1,8-cineole (52.0), camphor (10.4), *α*-pinene (6.0), camphene (4.7), *β*-pinene (3.9), myrcene (3.3)	[70]

P.p. = plant parts; a.p. = aerial parts; l. = leaves; f. = flowers; s. = stems; clt. = cultivated.

**Table 2:** The concentration of heavy metals (μg l^−1^) in waters of river measured in the two experimental sites (Acerra, C1; Castel Volturno, C2) (Maresca et al., 2018) [77].

	C1	C2
Cu	4743.46 ± 24.41 ^a^	10,812.52 ± 43.94 ^b^
Zn	4260.64 ± 11.02 ^a^	396,728.84 ± 1633.1 ^b^
Cd	1804.90 ± 9.38 ^a^	278,743.55 ± 685.84 ^b^
Pb	35.94 ± 4.50 ^a^	943.77 ± 22.53 ^b^

Values are presented as mean ± st. dev; numbers not accompanied by the same letter are significantly different at *p* < 0.05, using the post-hoc Student–Newman–Keuls test.

**Table 3:** Outline of the experimental design.

Heavy Metals Exposure	EO Treatment	Code
No exposure (without DMSO)	No essential oil	CTRL
No exposure (with DMSO)	No essential oil	CTRL-D
C1 Heavy Metals mix	No essential oil	C1
C2 Heavy Metals mix	No essential oil	C2
	Total EO extract treatments	
No exposure (with DMSO)	Total EO extract 0.16%	CTRL-TE16
C1 Heavy Metals mix	Total EO extract 0.16%	C1-TE16
C2 Heavy Metals mix	Total EO extract 0.16%	C2-TE16
No exposure (with DMSO)	Total EO extract 0.25%	CTRL-TE25
C1 Heavy Metals mix	Total EO extract 0.25%	C1-TE25
C2 Heavy Metals mix	Total EO extract 0.25%	C2-TE25
	Pure EOs treatments	
No exposure (with DMSO)	Camphor 0.16%	CTRL-CAM16
C1 Heavy Metals mix	Camphor 0.16%	C1-CAM16
C2 Heavy Metals mix	Camphor 0.16%	C2-CAM16
No exposure (with DMSO)	Camphor 0.25%	CTRL-CAM25
C1 Heavy Metals mix	Camphor 0.25%	C1-CAM25
C2 Heavy Metals mix	Camphor 0.25%	C2-CAM25
No exposure (with DMSO)	*β*-myrcene 0.16%	CTRL-MYR16
C1 Heavy Metals mix	*β*-myrcene 0.16%	C1-MYR16
C2 Heavy Metals mix	*β*-myrcene 0.16%	C2-MYR16
No exposure (with DMSO)	*β*-myrcene 0.25%	CTRL-MYR25
C1 Heavy Metals mix	*β*-myrcene 0.25%	C1-MYR25
C2 Heavy Metals mix	*β*-myrcene 0.25%	C2-MYR25
No exposure (with DMSO)	1,8-cineole 0.16%	CTRL-CIN16
C1 Heavy Metals mix	1,8-cineole 0.16%	C1-CIN16
C2 Heavy Metals mix	1,8-cineole 0.16%	C2-CIN16
No exposure (with DMSO)	1,8-cineole 0.25%	CTRL-CIN25
C1 Heavy Metals mix	1,8-cineole 0.25%	C1-CIN25
C2 Heavy Metals mix	1,8-cineole 0.25%	C2-CIN25

**Table 4:** Constituents of the EO of the flowering aerial parts of *Salvia fruticosa* collected in Sicily.

LRI ^a^	LRI ^b^	Compound	%	Identification ^c^
855	1318	1-Hexanol	0.03 ± 0.00	1, 2, 3
860	1344	(*Z*)-4-Hexen-1-ol	0.04 ± 0.00	1, 2
923	1007	Tricyclene	0.12 ± 0.00	1, 2
933	1025	*α*-Pinene	6.51 ± 0.27	1, 2, 3
950	1040	Camphene	8.69 ± 0.38	1, 2, 3
975	1080	*β*-Pinene	6.70 ± 0.21	1, 2, 3
980	1412	Oct-1-en-3-ol	0.13 ± 0.00	1, 2
989	1137	*β*-Myrcene	9.13 ± 0.38	1, 2, 3
1028	1172	1,8-Cineole (Eucalyptol)	17.56 ± 0.74	1, 2, 3
1057	1240	*γ*-Terpinene	1.38 ± 0.04	1, 2
1060	1470	(*E*)-Sabinene hydrate	0.30 ± 0.01	1, 2
1074	1493	(*Z*)-Sabinene hydrate	0.06 ± 0.00	1, 2
1089	1250	Terpinolene	0.45 ± 0.02	1, 2
1097	1513	*β*-Linalool	0.19 ± 0.00	1, 2, 3
1100	1368	*α*-Thujone	1.26 ± 0.04	1, 2
1106	1386	*β*-Thujone	2.11 ± 0.08	1, 2
1128	1564	(*E*)-*p*-2-Menthen-1-ol	0.13 ± 0.00	1, 2
1130	1515	Camphor	13.63 ± 0.54	1, 2, 3
1142	1818	*p*-Cymene-8-ol	0.03 ± 0.00	1, 2
1151	1640	Isoborneol	0.04 ± 0.00	1, 2
1162	1690	Borneol	3.69 ± 0.13	1, 2
1168	1592	Terpinene-4-ol	1.90 ± 0.06	1, 2
1180	1705	*α*-Terpineol	6.56 ± 0.27	1, 2, 3
1185	1342	(*E*)-1-Octenyl acetate	0.07 ± 0.00	1, 2
1265	1546	Bornyl acetate	1.63 ± 0.06	1, 2
1300	2167	Carvacrol	0.02 ± 0.00	1, 2
1366	1674	Isoledene	0.04 ± 0.00	1, 2
1432	1583	(*E*)-*β*-Caryophyllene	2.12 ± 0.09	1, 2
1439	1833	(*E*)-Geranylacetone	0.04 ± 0.00	1, 2
1448	1690	*α*-Humulene	0.27 ± 0.01	1, 2
1518	1796	(*E*)-Calamene	0.16 ± 0.00	1, 2
1532	1716	*δ*-Cadinene	0.78 ± 0.02	1, 2
1552	2019	Ledol	0.03 ± 0.00	1, 2
1567	2119	(*Z*)-3-Hexen-1-yl-benzoate	0.12 ± 0.00	1, 2
1569	2129	Spathulenol	0.18 ± 0.00	1, 2
1578	1960	Caryophyllene oxide	1.21 ± 0.04	1, 2,
1590	2027	Globulol	4.07 ± 0.15	1, 2
1592	2073	Viridiflorol	0.04 ± 0.00	1, 2
1648	2250	*α*-Eudesmol	0.05 ± 0.00	1, 2
2034	2603	Manool	3.01 ± 0.11	1, 2
2319	3203	Ferruginol	0.17 ± 0.00	1, 2
		Class of Compounds		
		Aliphatic alcohols	0.20 ± 0.00	
		Aliphatic esters	0.07 ± 0.00	
		Aromatic esters	0.12 ± 0.00	
		Monoterpene hydrocarbons	31.60 ± 1.30	
		Oxygenated monoterpenes	49.11 ± 1.93	
		Sesquiterpene hydrocarbons	3.41 ± 0.12	
		Oxygenated sesquiterpenes	5.58 ± 0.19	
		Oxygenated diterpenes	3.18 ± 0.11	
		Total	93.27 ± 3.65	

^a^ Linear Retention Index on a DB-5 MS column; ^b^ Linear Retention Index on a Supelcowax 10 column; ^c^ 1: linear retention index; 2: mass spectrum; 3: co-injection with authentic compound. Values are expressed as average of three measurements ± standard deviation.

## Data Availability

Data is contained within the article.
